# Serum long non-coding RNAs MALAT1, AFAP1-AS1 and AL359062 as diagnostic and prognostic biomarkers for nasopharyngeal carcinoma

**DOI:** 10.18632/oncotarget.17083

**Published:** 2017-04-13

**Authors:** Baoyu He, Jianchao Zeng, Wei Chao, Xiaoli Chen, Yujie Huang, Kaifeng Deng, Zhizhuo Huang, Jinwan Li, Meiyu Dai, Shaojun Chen, Haixin Huang, Shengming Dai

**Affiliations:** ^1^ Medical Science Laboratory, The Fourth Affiliated Hospital of Guangxi Medical University, Liuzhou, Guangxi 545005, China; ^2^ Department of Oncology, The Fourth Affiliated Hospital of Guangxi Medical University, Liuzhou, Guangxi 545005, China

**Keywords:** long non-coding RNA, serum, nasopharyngeal carcinoma, early diagnosis, prognostic prediction

## Abstract

Circulating RNAs in serum, plasma or other body liquid have emerged as useful and highly promising biomarkers for noninvasive diagnostic application. Herein, we aimed to establish a serum long non-coding RNAs (lncRNAs) signature for diagnosing nasopharyngeal carcinoma (NPC). In this study, we recruited a cohort of 101 NPC patients, 20 patients with chronic nasopharyngitis (CN), 20 EBV carriers (EC) and 101 healthy controls. qRT-PCR was performed with NPC cells and serum samples to screen a pool of 38 NPC-related lncRNAs obtained from the LncRNADisease database. A profile of three circulating lncRNAs (MALAT1, AFAP1-AS1 and AL359062) was established for NPC diagnosis. By Receiver Operating Characteristic (ROC) curve analysis, this three-lncRNA signature showed high accuracy in discriminating NPC from healthy controls (AUC = 0.918), CN (AUC = 0.893) or EC (AUC = 0.877). Furthermore, high levels of these three lncRNAs were closely related to advanced NPC tumor node metastasis stages and EBV infection. Serum levels of these three lncRNAs declined significantly in patients after therapy. Our present study indicates that circulating MALAT1, AFAP1-AS1 and AL359062 may represent novel serum biomarkers for NPC diagnosis and prognostic prediction after treatment.

## INTRODUCTION

Nasopharyngeal carcinoma (NPC) is the most prevailing head and neck tumor in southern China and Southeast Asia [1, 2]. Because of the poor accessibility for physical examination and the nonspecific symptoms at onset of disease, 75-90% of NPC cases are diagnosed at advanced stages [3, 4]. Moreover, the high relapse rate reduces the therapeutic effect and five-year survival rate of NPC patients [5]. Thus, it is essential to characterize highly sensitive and specific biomarkers for accurate early diagnosis of high-risk populations and assessment of NPC treatment effectiveness.

As important noninvasive method for early NPC screening, the detection of IgA antibodies against latent and lytic Epstein-Barr virus (EBV) proteins in serum has been routinely applied in the high-prevalence areas of NPC [6, 7]. However, specific antigen-antibody reaction with high reliability for NPC detection is still unavailable. Establishment of a NPC specific antibody profile that is suitable for clinical practice remains a challenging problem. Circulating cell-free EBV DNA in serum was reported as a diagnostic indicator for NPC presence and an independent prognostic factor for UICC staging in NPC [8–11]. However, accurate loads of viral nucleic acids among NPC patients and noncancerous controls remain uncharacterized. Moreover, significantly increased levels of these biomarkers also appeared among asymptomatic EBV carriers and patients with other diseases such as infectious mononucleosis and pharyngitis, which reduced the diagnostic specificity of these markers [12, 13]. In spite of this, detection of EBV-related biomarkers can be regarded as an alternate for NPC diagnosis.

Long non-coding RNAs (LncRNAs) are defined as non-coding RNAs longer than 200 nucleotides. Emerging evidence showed that lncRNAs were biologically significant in tumor initiation and progression [14–17]. Dysregulation of lncRNAs has been detected in tissue sections from multiple tumor types [18, 19]. Recent studies have confirmed that lncRNAs existed in serum or plasma at measurable levels with high stability, making them promising biomarkers [20]. For example, the serum-circulating lncRNA signature (lncRNA-LET, PVT1, PANDAR, PTENP1 and linc00963) can discriminate patients with clear cell renal cell carcinoma from healthy controls, and lncRNA-HULC can serve as a novel serum biomarker for diagnosis and prognostic prediction of gastric cancer [21, 22]. To date, a systematic selection of lncRNAs and evaluation of their efficacy as diagnostic and prognostic markers for NPC has not yet been reported.

In this study, we examined 38 NPC-related lncRNAs in four NPC cells (5-8F, 6-10B, CNE2 and CNE1) and 242 serum samples, including 101 NPC patients, 101 healthy controls (HC), 20 patients with chronic nasopharyngitis over five years (CN) and 20 asymptomatic EBV carriers (EC). After stepwise screening and validation, we established a three-lncRNA signature for early NPC detection. Moreover, we also assessed the prediction performance of lncRNAs identified for tumor progression and treatment efficacy. In the end, we investigated the biological function of AL359062, an uncharacterized lncRNA, *in vitro*.

## RESULTS

### LncRNAs MALAT1, AFAP1-AS1 and AL359062 were identified as potential biomarkers for NPC

Firstly, 38 NPC-related lncRNAs obtained from the LncRNADisease database were evaluated within four NPC cell lines (5-8F, 6-10B, CNE2 and CNE1) and non-tumorigenic nasopharyngeal epithelial cell (NP69). Based on those criteria listed in study design, 15 putative candidate lncRNAs were further detected in 20 pairs of sera from age- and sex-matched NPC patients and healthy controls. Eventually, three lncRNAs (MALAT1, AFAP1-AS1 and AL359062) remaining distinct in the NPC group were selected as potential biomarkers (Supplementary Table 1).

**Figure 1:**
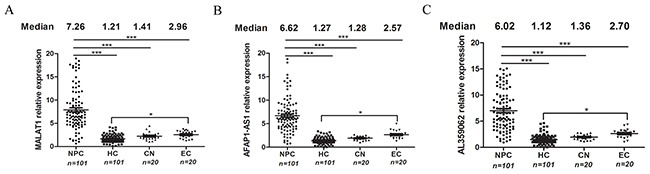
MALAT1, AFAP1-AS1 and AL359062 were identified as candidate lncRNAs for NPC detection Expression levels of circulating MALAT1 **(A)**, AFAP1-AS1 **(B)** and AL359062 **(C)** were measured with serum specimens obtained from NPC patients (n=101), HC (n=101), CN (n=20) and EC (n=20) via qPCR assay using the protocol described in the materials and methods. Each dot represents an individual and median expression levels of circulating lncRNAs were shown at the top. (*p < 0.05, **p < 0.005, ***p < 0.001).

We further validated these three lncRNAs in an independent cohort of 81 NPC, 81 HC, 20 CN and 20 EC subjects. Finally, the data from all screening stages were pooled and analyzed. Results showed that levels of MALAT1 (Figure 1A), AFAP1-AS1 (Figure 1B) and AL359062 (Figure 1C) significantly increased in NPC patients compared to HC, CN, or EC (all with *p* < 0.001). Therefore, these three lncRNAs were identified as potential biomarkers for NPC and were included in subsequent analyses.

### The three-lncRNA signature could discriminate early NPC patients from HC, CN or EC

**Figure 2:**
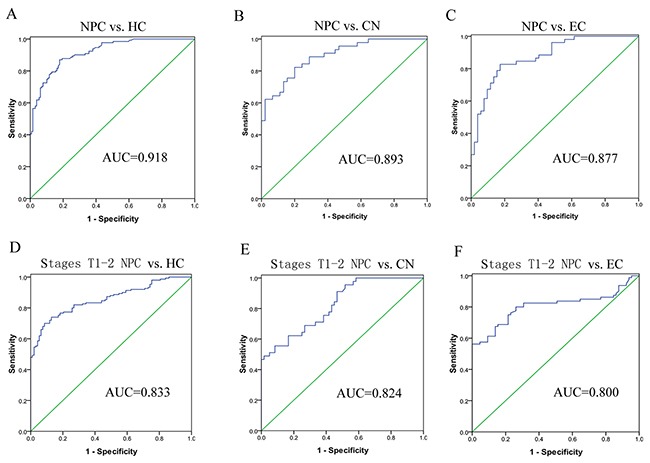
Diagnostic values of the three-lncRNA signature for early stage NPC detection ROC analysis of circulating three-lncRNAs signature was employed to discriminate NPC patients from HC **(A)**, CN **(B)** or EC **(C)**, respectively. The three-lncRNA signature showed high accuracy in discriminating early stage NPC (stages T1-2) from HC **(D)**, CN **(E)** or EC **(F)**.

Receiver operating characteristic (ROC) analysis revealed the discriminative abilities for each single lncRNA (Table 1). Then, a stepwise selection model demonstrated that the combination of MALAT1, AFAP1-AS1 and AL359062 provided the greatest predictive ability, especially the sensitivity, with an area under the curve (AUC) of 0.918 for NPC vs. HC (Figure 2A), 0.893 for NPC vs. CN (Figure 2B) and 0.877 for NPC vs. EC (Figure 2C). Diagnostic performances of serum lncRNAs for different models were listed in Table 1.

**Table 1:** Diagnostic performances of serum MALAT1, AFAP1-AS1 and AL359062 for NPC detection

LncRNAs	NPC vs. HC				NPC vs. CN				NPC vs. EC			
	AUC	95%CI	SEN	SPE	AUC	95%CI	SEN	SPE	AUC	95%CI	SEN	SPE
MALAT1	0.645	0.589-0.686	0.664	0.889	0.636	0.575-0.696	0.612	0.852	0.566	0.515-0.612	0.525	0.889
AFAP1-AS1	0.665	0.598-0.702	0.640	0.838	0.625	0.563-0.685	0.590	0.822	0.620	0.572-0.580	0.592	0.819
AL359062	0.610	0.552-0.672	0.677	0.889	0.526	0.475-0.583	0.512	0.833	0.596	0.538-0.656	0.555	0.898
MALAT1+AFAP1-AS1	0.702	0.652-0.751	0.782	0.874	0.711	0.662-0.765	0.708	0.855	0.698	0.642-0.762	0.715	0.856
MALAT1+AL359062	0.721	0.674-0.782	0.792	0.876	0.700	0.641-0.766	0.725	0.845	0.742	0.686-0.793	0.735	0.825
AFAP1-AS1+AL359062	0.755	0.701-0.821	0.791	0.885	0.698	0.642-0.766	0.799	0.869	0.763	0.687-0.822	0.716	0.889
MALAT1+AFAP1-AS1+AL359062	0.918	0.886-0.949	0.933	0.868	0.893	0.831-0.956	0.902	0.802	0.877	0.813-0.942	0.899	0.813

AUC, area under curve; 95%CI, 95% confidence intervals; SEN, sensitivity; SPE, specificity

It is noteworthy that this three-lncRNA signature showed high accuracy in discriminating early stage NPC (T1-2) from HC (AUC = 0.833) (Figure 2D), CN (AUC = 0.824) (Figure 2E) or EC (AUC = 0.800) (Figure 2F), suggesting that the signature could predict NPC at relatively early stages. However, the efficacy of this panel in early NPC diagnosis needs to be examined in a larger cohort.

### High MALAT1, AFAP1-AS1 and AL359062 levels were related to EBV infection

**Figure 3:**
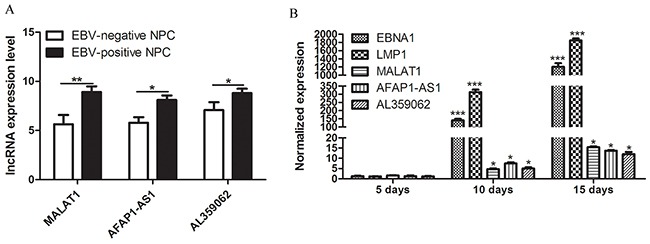
High MALAT1, AFAP1-AS1 and AL359062 levels were related to EBV infection **(A)** qRT-PCR assay examined the differences of expression levels of circulating MALAT1, AFAP1-AS1 and AL359062 between EBV-positive NPC group and EBV-negative NPC group. **(B)** Expression levels of MALAT1, AFAP1-AS1 and AL359062 were detected in culture supernatant of EBV infected NP69 on Day 5, 10, and 15 of post-infection. EBV genes EBNA1 and LMP1 were used to measure the level of viral replication. (*p < 0.05, **p < 0.005, ***p < 0.001).

Additionally, we have noticed an interesting result that levels of circulating MALAT1, AFAP1-AS1 and AL359062 in EC statistically increased compared to HC (Figures 1A-1C). Moreover, EBV-positive NPC group had higher levels of circulating lncRNAs than EBV-negative NPC group (*p* < 0.05) (Figure 3A). Further, we conducted the infection of NP69 cell with EBV particles produced by B95-8 cells. Along with the persistent infection, levels of MALAT1, AFAP1-AS1 and AL359062 significantly increased in the infected NP69 cell (data not shown) and culture supernatant (Figure 3B).

### High levels of serum MALAT1, AFAP1-AS1 and AL359062 were closely associated with advanced NPC tumor node metastasis (TNM) stages

**Figure 4:**
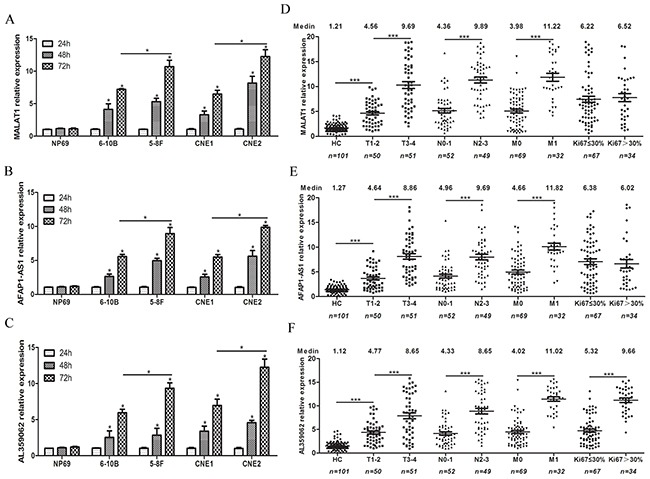
High levels of serum MALAT1, AFAP1-AS1 and AL359062 were closely associated with advanced NPC tumor node metastasis (TNM) stages Suspended MALAT1 **(A)**, AFAP1-AS1 **(B)** and AL359062 **(C)** were detected in the culture supernatant of four NPC cells and NP69 cell incubated for 24 h, 48 h and 72 h. Data represent the mean ± standard deviation from three independent assays. Levels of circulating MALAT1 **(D)**, AFAP1-AS1 **(E)** and AL359062 **(F)** were measured in sera from NPC patients of different TNM subgroups and histological types. (*p < 0.05, **p < 0.005, ***p < 0.001).

Firstly, we detected the levels of MALAT1, AFAP1-AS1 and AL359062 in supernatant of NPC cells and NP69 cell. As results showed, the lncRNAs expression levels in the supernatant of these NPC cells increased in a time-dependent manner while no significant change was observed in that of NP69 cell (Figures 4A-4C). Furthermore, higher levels of lncRNAs appeared in the supernatant of 5-8F (highly invasive) and CNE2 (poorly differentiated) cells compared to that in 6-10B (poorly invasive) and CNE1 (highly differentiated) cells, respectively (Figures 4A-4C).

Due to the close correlations with the malignancy of NPC cells, MALAT1, AFAP1-AS1 and AL359062 were further analyzed in serum samples from NPC patients at different stages of disease progression. Results revealed that high levels of circulating MALAT1, AFAP1-AS1 and AL359062 were closely related to advanced NPC TNM stages. Specifically, the levels of circulating MALAT1 (Figure 4D), AFAP1-AS1 (Figure 4E) and AL359062 (Figure 4F) of NPC patients of T3-4 subgroups, N2-3 subgroups and M1 subgroup were remarkably higher than that of T1-2 subgroups, N0-1 subgroups and M0 subgroup (all with *p* < 0.001), respectively. In addition, we also found a noticeable correlation between AL359062 level and Ki67 labeling index (Figure 4D). Spearman correlation coefficients (*r*-values) and the corresponding *p*-values for the associations between lncRNAs levels and tumor progression were listed in Table 2.

**Table 2:** Spearman's relative testing of significant associations between levels of serum MALAT1, AFAP1-AS1 and AL359062 and tumor progression

LncRNAs	T1-2/T3-4 (50/51)		N0-1/N2-3 (52/49)		M0-M1 (69/32)		Ki67≤30%/Ki67>30% (67/34)	
	r-value	p-value	r-value	p-value	r-value	p-value	r-value	p-value
MALAT1	0.566	< 0.001	0.571	< 0.001	0.599	< 0.001	0.080	0.479
AFAP1-AS1	0.398	< 0.001	0.616	< 0.001	0.427	< 0.001	0.006	0.956
AL359062	0.431	< 0.001	0.571	< 0.001	0.625	< 0.001	0.637	< 0.001

### Serum MALAT1, AFAP1-AS1 and AL359062 levels declined after therapy in NPC patients

**Figure 5:**
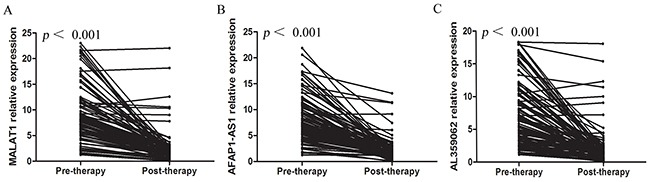
Serum MALAT1, AFAP1-AS1 and AL359062 levels declined after therapy in NPC patients The Wilcoxon signed-rank test results showed that levels of circulating MALAT1 **(A)**, AFAP1-AS1 **(B)** and AL359062 **(C)** were all reduced (all with *p* < 0.001) in post-therapeutic sera compared to the pre-therapeutic group. Each line represents one individual patient.

We compared the levels of these three lncRNAs in pre- and post-therapeutic serum samples of NPC patients. We found the levels of serum MALAT1 (Figure 5A), AFAP1-AS1 (Figure 5B) and AL359062 (Figure 5C) were all dramatically reduced or even diminished after therapy in most patients. However, there were several cases with no change in lncRNAs levels after treatment. Clinical examination reported that these cases either suffered relapse or had residual NPC tumor in lymphnodes or metastasis during follow-up (Supplementary Table 2). The results suggested that these markers may be of value to monitor the efficacy of therapy. And high-level serum MALAT1, AFAP1-AS1 and AL359062 after therapy are associated with poor prognosis in NPC patients.

### AL359062 knockdown suppress NPC cell metastasis, invasion and proliferation *in vitro*

**Figure 6:**
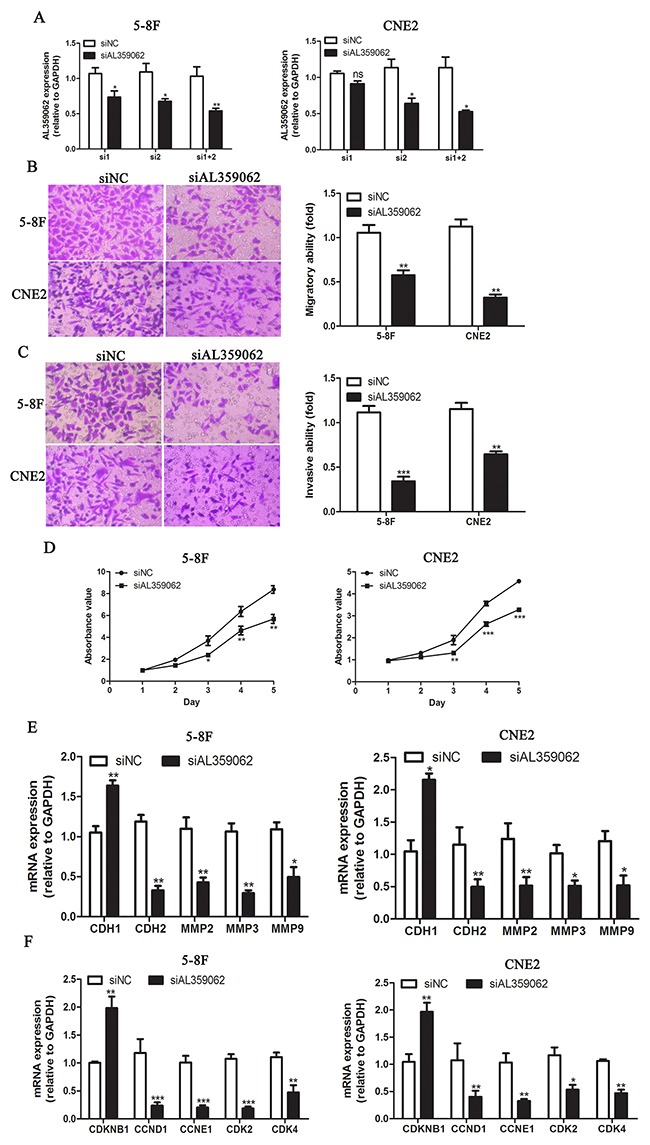
AL359062 knockdown suppress NPC cell metastasis, invasion and proliferation *in vitro* **(A)**. siRNA1+2 efficiently suppressed AL359062 expression compared with the siNC in 5-8F and CNE2 cells by qRT-PCR. **(B-C)**. AL359062 knockdown inhibited NPC cell migratory and invasive abilities as measured by transwell migration assay (B) and matrigel invasion assay (C). **(D)**. CCK-8 assay was applied to detect the effect of AL359062 knockdown on NPC cell viability. **(E-F)**. Forty-eight hours after AL359062 knockdown, total RNA was extracted and mRNA expression levels of metastasis and invasion-related genes CDH1, CDH2, MMP2, 3 and 9 (E), and cell cycle-related genes CDKN1B, CCND1, CCNE1, CDK2 and 4 (F) were measured by qRT-PCR. The graph summarizes the data from three independent experiments. (*p < 0.05, **p < 0.005, ***p < 0.001).

Previous studies have demonstrated the roles of MALAT1 and AFAP1-AS1 in initiation and progression of NPC [14–17]. However, the functional role of AL359062 was poorly understood. Here, we synthesized siRNA sequence targeting AL359062. qRT-PCR results showed that the mixture of siRNA1 and siRNA2 significantly knocked down AL359062 expression compared with the scrambled negative control (siNC) in 5-8F and CNE2 cell (Figure 6A). The AL359062 siRNA (siAL359062) significantly reduced the migratory activity of NPC cells in Boyden chamber assays (Figure 6B). Similarly, treatment with siAL359062 suppressed the invasiveness of NPC cells in Matrigel-coated Boyden chamber assays (Figure 6C). qRT-PCR results showed that siAL359062 increased the mRNA expression level of CDH1 (encoding E-cadherin) and decreased CDH2 (encoding N-cadherin), MMP2, 3 and 9 (Figure 6E).

Cell proliferation was measured by using the CCK-8 assay for five days. On Day 3, 4 and 5, the number of viable cells was significantly decreased in siAL359062 group compared with the controls (Figure 6D). qRT-PCR results showed that siAL359062 increased the mRNA expression level of CDKN1B (encoding cyclin-dependent kinase inhibitor 1B) and decreased CCND1 (encoding cyclin D1), CCNE1 (encoding cyclin E1), CDK2 and 4 (encoding cyclin dependent kinase 2 and 4, respectively) (Figure 6F).

## DISCUSSION

Circulating lncRNAs in serum or plasma have good potential to serve as diagnostic or prognostic markers in diseases, especially in some types of cancer, as they are stable, accessible and closely related to the disease [23–25]. In this study, we comprehensively evaluated the circulating lncRNAs levels in a large cohort of sera from 101 NPC patients. The circulating lncRNA panel (MALAT1, AFAP1-AS1 and AL359062) identified by a stepwise selection model was highly indicative for the NPC detection. Further Spearman's relative test indicated high levels of circulating MALAT1, AFAP1-AS1 and AL359062 were closely correlated with advanced TNM stages. Moreover, significantly increased levels of MALAT1, AFAP1-AS1 and AL359062 appeared in the culture supernatant of highly invasive NPC cells. Based on these results, we speculated that serum lncRNAs may be derived from tumor cells, especially those highly malignant and invasive tumor cells, and thus can reflect the status of tumor progression. These results may explain why the decreased lncRNAs levels appeared in the majority of NPC patients after therapy. Taken together, it was reasonable to suggest that serum MALAT1, AFAP1-AS1 and AL359062 can precisely evaluate the therapeutic outcome and predict the prognosis for NPC. Up to now, this is the first time to systematically characterize circulating lncRNAs in serum as diagnostic and prognostic biomarkers for NPC.

Persistently latent EBV infection has been found among the majority of NPC patients, indicating its etiological role in initiation and progression of NPC [26–28]. Here, we found levels of MALAT1, AFAP1-AS1 and AL359062 significantly increased in NP69 cell after EBV infection. Our earlier research has found LOC553103, a novel human lncRNA that was down-regulated in the EBV-positive NPC cell line (C666-1). Further luciferase assay confirmed that EBV-miR-BART6-3p directly targeted LOC553103 [29]. In fact, an increasing number of human non-coding RNAs, including miRNAs and lncRNAs, were reported to be regulated by genes of EBV. For instance, Chen et al [30] demonstrated that EBV encoded-latent membrane protein-1 increased miR-1 expression and thus promoting tumor growth and metastasis by targeting K-ras in NPC cells. In this study, MALAT1, AFAP1-AS1 and AL359062 showed a close correlation with EBV infection. However, it is still undetermined how EBV infection regulates signaling pathways to promote expression levels of these three lncRNAs. The recognition of cross-talk between EBV infection and lncRNAs would provide novel evidence for the influence of EBV infection on carcinogenesis.

Recently, an increasing number of lncRNAs have been reported to be associated with tumorigenesis and the progression of NPC [17, 31, 32]. Some lncRNAs, such as HOTAIR [31, 33], H19 [34], NEAT1 [35], HNF1A-AS [36], ANRIL [32], are reported to be oncogenic molecules in NPC, while GAS [37] and MEG3 [38] lncRNAs act as tumor suppressors. MALAT1 was originally identified as a prognostic marker for non-small cell lung cancer, and then was found upregulated in a range of tumor types [39–41]. MALAT1 promotes initiation and progression of NPC by acting as a sponge of miR-25 [42] or miR-1 [43]. AFAP1-AS1 is a newly identified lncRNA [44]. High expression of AFAP1-AS1 is associated with metastasis and poor prognosis in NPC patients [14]. Therefore, it might be feasible for AFAP1-AS1 to be a diagnostic biomarker and prognostic factor. To date, AL359062 has only been studied in NPC tissues [45]. The expression level of AL359062 was negatively correlated with the prognosis of NPC patients. Here, we found AL359062 knockdown suppressed NPC cell metastasis, invasion and proliferation *in vitro*. Further qRT-PCR assay showed that AL359062 affected the expression of genes related to metastasis, invasion and cell cycle. These genes may be involved in the process of AL359062-induced carcinogenesis. However, it remains unclear how AL359062 regulates signaling pathways to enhance cancer cell invasion, migration and proliferation.

To summarize, we have successfully established a distinctive serum three-lncRNA signature (MALAT1, AFAP1-AS1 and AL359062) for NPC detection through stringent step-by-step selection procedures. Simultaneously, serum MALAT1, AFAP1-AS1 and AL359062 have potential clinical values for forecasting tumor progression and predicting the therapeutic efficacy for NPC. In spite of our efforts, it is still uncertain if this signature is only specific for NPC. Thus, additional studies will be required to further examine the expression changes of the three lncRNAs in other tumors.

## MATERIALS AND METHODS

### Study design

Here, a three-phase method was used to systematically investigate the expression of NPC-specific lncRNAs.

#### Initial phase

we entered the keyword “nasopharyngeal carcinoma” as search term in the lncRNADisease database (http://www.cuilab.cn/lncrnadisease), and 38 differentially expressed lncRNAs were selected.

#### Training phase

Expression profiles of 38 lncRNAs were firstly verified in four NPC cell lines as well as non-tumorigenic nasopharyngeal epithelial cell. The criteria for cell-level investigation of these selected candidates were: (i) the quantification cycle values ≤ 30; (ii) lncRNAs expressed twofold higher in NPC cells compared to NP69 cell. Subsequently, the verified lncRNAs was further examined in serum samples from 20 NPC and 20 HC. The criteria of serum-level investigation for selected candidates were: (i) the quantification cycle values ≤ 35 to gain credibility; (ii) the detectable rate > 80%, meaning over 16 of 20 cases of samples were detectable; (iii) the expression levels were statistically higher in NPC samples compared to HC.

#### Validation phase

Three candidate lncRNAs in training phase were further validated in another independent cohorts of serum samples including 81 NPC, 81 HC, 20 CN and 20 EC subjects. The data from all above NPC patients (101), HC (101), CN (20) and EC (20) were used to construct the diagnostic signature.

### Patients and sera collection

The sera from a total of 242 participants, including 101 NPC patients, 101 HC, 20 CN and 20 EC, were selected into this study. Table 3 lists the clinical characteristics and pathological information of the subjects. Patients and controls were recruited from October, 2014 to May, 2016. The TNM stage was established in accordance with the seventh edition of AJCC/IUCC Staging System. One hundred and one NPC patients were all newly diagnosed with primary NPC and randomly divided into two groups: training set (20 patients) and validation set (81 patients). Venous blood samples were collected from patients prior to any radiotherapy and chemotherapy treatment. A total of 101 individuals attending to the regular health examination served as age- and sex-matched healthy controls. The study was approved by the Institutional Ethical Review Board from the Fourth Affiliated Hospital of Guangxi Medical University (IRB No. PJ201401), and written informed consent was obtained from all patients and healthy volunteers. All serum samples were processed within 4 h after blood drawing. Briefly, whole blood was drawn into blood vessels without adding anticoagulants and separated to get serum by centrifugation at 1,500 g for 15 min.

**Table 3:** Clinical characteristics of patients and controls

Characteristics	HC	CN	EC	NPC
Number of cases	101	20	20	101
Gender (male/female)	60/41	13/7	11/9	60/41
Age (years; mean ± SD)	43.1±8.9	43.7±11.4	45.6±14.8	45.8±11.1
Tumor status (T1/T2/T3/T4)				25/25/27/24
lymph nodes status (N0/N1/N2/N3)				18/34/30/19
Metastasis (M0/M1)				69/32
Cancer stage (I/II/III/IVAB)				15/27/35/24
Ki67 index ≤/>30%				67/34

### Treatment

Among these 101 NPC patients, 69 without distant organ metastasis received an image-guided intensity modulated radiotherapy (IMRT) using a uniform protocol as previously described [46, 47]. Specifically, a total dose of 68.2-72.6 Gy in 31-33 fractions at 2.20 Gy/fraction to the PTVs of GTV-T and GTV-N, 61.5-67.65 Gy in 30–33 fractions at 2.05 Gy/fraction to the PTV of CTV-1, 55.5-59.2 Gy in 30–32 fractions at 1.85 Gy/fraction to PTVs of CTV-2 and CTV-N were prescribed in the treatment. The other 32 patients with stage M1 disease were provided with a comprehensive therapy method including cisplatin-based chemotherapy and primary radiation therapy. Paired sera of pre- and post- treatment from the 101 NPC patients were collected.

### Cell culture and siRNA

All cell lines were obtained from Shanghai Institutes for Biological Sciences (Shanghai, China). 6-10B, 5-8F, CNE1, CNE2 and B95-8 cells were cultured in DMEM (Gibco) or RPMI 1640 medium (Gibco) containing 10% fetal bovine serum (Gibco) and 1% penicillin-streptomycin at 37°C in a humidified environment with 5% CO_2_. NP69 cell was cultured in the Keratinocyte-SFM medium which contained epidermal growth factor 1-53 (EGF1-53) and bovine pituitary extract (BPE) (Gibco). Supernatants were collected for measurement of extracellular lncRNAs 24 h, 48 h and 72 h after subculture. Culture supernatants were purified by centrifugation at 2,000 g for 15 min to remove cell remnants. The sequences of the AL359062 siRNA1 and siRNA2 were 5’-GCACAAATCAAAGGCCAAT-3’ and 5’-GCCTGACACCTTCCCGATAC-3’ respectively, which were provided by TaKaRa.

### EBV infection

About one week after treatment with serum starvation, the B95-8 cell lysed and the EBV particles were fully released. First, culture suspension were clarified by centrifugation of 4,000 g at 4 °C for 30 min to remove cell debris. Then, the EBV particles were precipitated by centrifugation of 12,000 g at 4 °C for 2.5h. Virus precipitate was dissolved in fresh serum-free medium. The concentrated EBV suspension was filtered through a 0.22-μm pore-size filter and then frozen at -80°C prior to infection. EBV infection of NP69 cell was accomplished by a newly-developed approach based on the cell-free EBV infection as previously reported [48, 49]. Briefly, NP69 cell was grown to 70-80% confluence in a six-well tissue culture dish and then incubated with 1 ml virus concentrate for 10 h at 37°C in 5% CO_2_ with saturated humidity. Then, the virus concentrate were replaced with 3.0 ml fresh Keratinocyte-SFM medium. The infection was totally repeated for three times with a two-day interval. After then, cells and the supernatant were collected separately for RNA isolation on Day 5, 10, 15, respectively.

### RNA extraction and qRT-PCR

RNAiso Plus reagent (TaKaRa) and RNAiso Blood reagent (TaKaRa) were applied for RNA extraction from culture supernatant and serum, respectively. PrimeScript™ RT Reagent Kit (TaKaRa) with gDNA Eraser was incubated with 1μg RNA for reverse transcription. SYBR^®^ Premix Ex Taq™ II (TaKaRa) was used for fluorescence quantitative PCR assay. Glyceraldehyde-3-phosphate dehydrogenase (GAPDH) was used as an endogenous control to normalize the data because it is stably expressed in serum or plasma. The entire process was conducted in accordance with the manufacturer's instructions. All qRT-PCR reactions were performed on ABI 7500 Real-Time PCR System (Applied Biosystems, CA, USA). The expression levels of lncRNAs were calculated using 2^−ΔΔCt^ method. All the specific primers in this study were synthesized by TaKaRa and listed in the Supplementary Table 3.

### Transwell migration assay and invasion assay

The migration assay was performed using 24-well transwell inserts with an 8 μm pore size (Corning). The invasion assay was performed by precoating the transwell inserts with Matrigel Basement Membrane Matrix (BD Biosciences). In brief, transient transfection cells suspended in serum-free medium were seeded in the upper chamber and were allowed to transmigrate towards the bottom chamber, containing medium with 15% FBS for 36 h. The membrane inserts were then fixed with 4% paraformaldehyde and stained with 1% gentian violet. Images were captured from each membrane, and the number of invasive cells was counted under a microscope.

### Cell viability CCK-8 assay

Changes in cell viability were determined by adding 20 μl CCK-8 reagent (Dojindo Molecular Technologies) to each well on Day 0, 1, 2, 3, 4 and 5. Then light absorbance was measured at a wavelength of 450 nm. Experiments were performed in sextuplicate and repeated once.

### Statistical analysis

Statistical analysis was performed using the SPSS 17.0 software version (SPSS Inc.). A Student's t-test was used to evaluate differences in the expression of the selected lncRNAs in NPC cell lines and sera from the NPC patients and corresponding controls. *p* values < 0.05 were considered statistically significant, and all statistical tests were two-sided. The sensitivity, specificity and AUC for the lncRNA identified were determined using a ROC analysis. Significant associations between lncRNAs levels and clinic pathological parameters were assessed with a Spearman's relative test. The Wilcoxon test was used to compare the paired serum samples obtained before and after treatment. The figures were partially drawn by GraphPad Prism 5.0 software (Graphpad Software Inc.).

## SUPPLEMENTARY MATERIALS TABLES








