# Supplementary material for: Serum long non-coding RNAs MALAT1, AFAP1-AS1 and AL359062 as diagnostic and prognostic biomarkers for nasopharyngeal carcinoma
Source: Oncotarget. 2017 Apr 13;8(25):41166–77. doi: 10.18632/oncotarget.17083 (PMC5522198; doi:10.18632/oncotarget.17083)
Supplement: Supplementary file 1 [file oncotarget-08-41166-s001.pdf]

Serum long non-coding RNAs MALAT1, AFAP1-AS1 and AL359062 as diagnostic and prognostic biomarkers for nasopharyngeal carcinoma

SUPPLEMENTARY MATERIALS

Supplementary Table 1: Statistical expression results of 38 lncRNAs in four NPC cells and 20 cases of serum samples.

See Supplementary File 1

Supplementary Table 2: Levels of serum MALAT1, AFAP1-AS1 and AL359062 in NPC patients before and after treatment.

See Supplementary File 2

Supplementary Table 3: Primer sequences used for qRT-PCR.

See Supplementary File 3
