# Supplementary material for: Serum long non-coding RNAs MALAT1, AFAP1-AS1 and AL359062 as diagnostic and prognostic biomarkers for nasopharyngeal carcinoma
Source: Oncotarget. 2017 Apr 13;8(25):41166–77. doi: 10.18632/oncotarget.17083 (PMC5522198; doi:10.18632/oncotarget.17083)
Supplement: Supplementary file 2 [file oncotarget-08-41166-s002.doc]

**Supplemental Data**

**Supplemental Table S1. Statistical expression results of 38 lncRNAs in four NPC cells and 20 cases of serum samples.**

| **lncRNAs** | **Regulation and fold change in 5-8F/6-10B** | | **Regulation and fold change in CNE2/CNE1** | | **Detectable rate and *p* value**  **in 20 cases of sera** |
| --- | --- | --- | --- | --- | --- |
| H19***** | up**/**up | 23.25/3.12 | up**/**up | 35.85/5.65 | 0.95/0.125 |
|
| NEAT1***** | up**/**up | 10.25/7.32 | up**/**up | 11.25/9.36 | 1/0.102 |
|
| HNF1A-AS1***** | up**/**up | 6.32/6.02 | up**/**up | 4.25/2.36 | 1/0.087 |
|
| HOTAIR***** | up**/**up | 2.36/2.66 | up**/**up | 4.36/3.22 | 1/0.076 |
|
| MALAT1***** | up**/**up | 9.35/5.32 | up**/**up | 14.36/7.27 | 1/0.004 |
|
| LINCRNA-P21 | up**/**up | 2.45/2.66 | up/down | 4.25/3.66 |  |
|
| GAS 5 | up**/**up | 4.25/3.52 | up**/**up | 2.33/1.25 |  |
|
| AFAP1-AS1***** | up**/**up | 8.76/4.23 | up**/**up | 9.36/4.25 | 1/0.013 |
|
| LET | down**/**down | 4.12/4.36 | down**/**down | 1.22/1.56 |  |
|
| LOC401317 | down**/**down | 2.14/2.52 | up**/**down | 2.35/4.23 |  |
|
| NAG7 | up**/**up | 5.36/1.02 | up**/**up | 2.25/2.31 |  |
|
| CCAT2 | up**/**up | 3.25/1.52 | up**/**up | 1.32/3.22 |  |
|
| LOC84740***** | up**/**up | 2.35/2.25 | up**/**up | 3.25/2.25 | 0.9/0.096 |
|
| ENST00000498296***** | up**/**up | 2.11/2.45 | up**/**up | 2.35/2.59 | 1/0.125 |
|
| AL359062***** | up**/**up | 5.66/5.02 | up**/**up | 5.22/2.32 | 1/0.014 |
|
| ENST00000438550***** | up**/**up | 3.02/2.25 | up**/**up | 2.53/2.69 | 0.85/0.088 |
|
| AF086415 | up**/**up | 2.33/1.28 | up**/**up | 1.25/2.36 |  |
|
| AK095147 | up**/**up | 1.52/1.66 | up**/**down | 2.03/2.66 |  |
|
| RP1-179N16.3 | down**/**down | 2.22/1.36 | down**/**down | 2.59/2.69 |  |
|
| MUDENG | down**/**down | 1.65/2.55 | down**/**down | 2.36/1.05 |  |
|
| AK056098 | down**/**down | 5.11/1.05 | down**/**down | 3.21/2.23 |  |
|
| AK294004 | down**/**down | 2.34/2.04 | up**/**down | 2.25/1.06 |  |
|
| LNC-C22ORF32-1***** | up**/**up | 4.35/3.22 | up**/**up | 2.36/2.54 | 0.75/0.459 |
|
| LNC-ZNF674-1***** | up**/**up | 3.22/2.85 | up**/**up | 4.25/4.33 | 0.85/0.254 |
|
| LNC-TLR4-1***** | up**/**up | 2.54/2.66 | up**/**up | 3.25/3.66 | 0.8/0.075 |
|
| LNC-BCL2L11-3***** | up**/**up | 2.68/3.02 | up**/**up | 4.52/4.68 | 1/0.173 |
|
| LNC-AL355149.1-1***** | up**/**up | 3.06/3.51 | up**/**up | 3.65/3.85 | 0.95/0.089 |
|
| ENST00000411815 | up**/**up | 1.02/1.66 | up**/**up | 2.35/4.25 |  |
|
| ENST00000429469 | up**/**up | 1.33/1.55 | up**/**up | 1.25/2.36 |  |
|
| ENST000000440518 | up**/**up | 2.36/1.22 | up**/**up | 2.35/2.46 |  |
|
| ENST00000443373 | up**/**up | 4.24/3.56 | up**/**down | 2.36/2.55 |  |
|
| ENST00000457799 | up**/**up | 5.31/1.32 | up**/**up | 3.25/2.22 |  |
|
| ENST00000513638 | up**/**up | 1.08/1.69 | up**/**up | 1.02/1.65 |  |
|
| ENST00000514571 | up**/**up | 4.23/1.56 | up**/**up | 2.35/2.01 |  |
|
| ENST00000565929 | up**/**up | 1.55/2.36 | up**/**up | 2.55/1.69 |  |
|
| ENST00000566575 | up**/**up | 4.36/5.36 | up**/**up | 1.65/1.86 |  |
|
| UC010ZYE | up**/**up | 2.36/4.25 | up**/**up | 2.36/3.22 |  |
|
| LINC00592 | up**/**up | 5.33/2.11 | up**/**up | 2.22/1.03 |  |
|
|

lncRNA* were the lncRNA candidates identified from cell-level screening process.

lncRNA* were the potential lncRNA biomarkers identified in training phase that need further validation in large-scale serum samples.
