# Supplementary material for: Serum long non-coding RNAs MALAT1, AFAP1-AS1 and AL359062 as diagnostic and prognostic biomarkers for nasopharyngeal carcinoma
Source: Oncotarget. 2017 Apr 13;8(25):41166–77. doi: 10.18632/oncotarget.17083 (PMC5522198; doi:10.18632/oncotarget.17083)
Supplement: Supplementary file 3 [file oncotarget-08-41166-s003.doc]

**Supplemental Table S2. Levels of serum MALAT1, AFAP1-AS1 and AL359062 in NPC patients before and after treatment.**

| Cases  N=101 | Gender | Age  (years) | Disease stage | MALAT1 | | AFAP1-AS1 | | AL359062 | | Clinical findings during follow-up |
| --- | --- | --- | --- | --- | --- | --- | --- | --- | --- | --- |
|  |  |  |  | Before | After | Before | After | Before | After |  |
| 1 | F | 47 | T3N3M1 | 6.223001 | 1.182829 | 7.130907 | 1.151510 | 3.621232 | 1.889374 |  |
| 2 | M | 51 | T3N3M1 | 6.450050 | 1.633769 | 5.916877 | 1.130099 | 6.610212 | 2.401793 |  |
| 3 | M | 66 | T3N2M1 | 5.356149 | 2.150848 | 7.596646 | 1.164286 | 4.289186 | 1.966816 |  |
| 4 | M | 54 | T3N2M1 | 6.193872 | 1.140072 | 8.050837 | 1.125721 | 4.820830 | 1.144100 |  |
| 5 | M | 28 | T3N2M1 | 5.817269 | 1.126459 | 4.334427 | 1.346696 | 12.008730 | 1.121269 |  |
| 6 | F | 49 | T3N0M0 | 7.023224 | 1.136071 | 5.090305 | 1.125861 | 18.212280 | 1.140908 |  |
| 7 | M | 29 | T3N2M0 | 2.274540 | 1.155232 | 4.019632 | 2.831513 | 5.234521 | 1.155237 |  |
| 8 | F | 43 | T1N2M0 | 7.620864 | 1.891189 | 6.352839 | 1.492361 | 7.904469 | 1.123459 |  |
| 9 | F | 37 | T1N0M0 | 17.047970 | 1.164296 | 5.349735 | 1.151109 | 9.026048 | 1.136275 |  |
| 10 | F | 58 | T1N0M0 | 11.668690 | 1.898174 | 7.178547 | 0.002900 | 11.735070 | 5.221382 |  |
| **11** | M | 52 | **T2N2M0** | **10.707920** | **10.321600** | **9.063197** | **9.162278** | **18.322640** | **18.075049** | **Recurrence and metastasis** |
| **12** | M | 57 | **T3N3M1** | **17.556518** | **18.183116** | **14.707058** | **11.406578** | **13.301169** | **11.439402** | **Residual lymph nodes** |
| 13 | M | 68 | T1N1M0 | 7.713521 | 1.358451 | 18.738330 | 1.206292 | 15.457150 | 1.580985 |  |
| 14 | M | 48 | T2N0M0 | 7.347512 | 1.436133 | 5.768173 | 1.663152 | 8.679236 | 3.278564 |  |
| 15 | M | 24 | T2N0M0 | 5.678876 | 1.168786 | 5.394699 | 1.999451 | 10.160730 | 1.166372 |  |
| 16 | F | 70 | T2N1M0 | 1.645975 | 0.127140 | 7.013073 | 3.258205 | 6.561707 | 2.435908 |  |
| 17 | M | 34 | T3N2M1 | 7.199354 | 1.369045 | 10.329170 | 3.794366 | 6.567367 | 3.704545 |  |
| 18 | F | 53 | T1N1M0 | 6.265639 | 1.146003 | 6.712237 | 1.673687 | 11.979430 | 1.382486 |  |
| 19 | M | 56 | T3N1M0 | 8.316611 | 1.252337 | 7.265321 | 1.799145 | 11.227960 | 1.253521 |  |
| 20 | M | 63 | T3N2M1 | 6.338065 | 1.376855 | 9.453032 | 2.067957 | 4.942392 | 2.494296 |  |
| 21 | M | 48 | T3N2M0 | 1.474821 | 0.145933 | 7.898164 | 1.766516 | 4.022366 | 1.251095 |  |
| 22 | M | 65 | T3N0M0 | 4.925385 | 1.169283 | 8.356483 | 1.408473 | 6.235283 | 2.619493 |  |
| 23 | M | 56 | T4N3M1 | 5.691482 | 1.593841 | 5.537306 | 2.864542 | 3.179419 | 1.186116 |  |
| 24 | M | 68 | T4N3M1 | 5.142076 | 1.133347 | 9.493976 | 1.553498 | 6.265966 | 2.622175 |  |
| 25 | F | 51 | T4N2M1 | 8.173781 | 1.675210 | 7.943072 | 1.154993 | 7.633199 | 1.291262 |  |
| 26 | M | 57 | T4N3M1 | 7.866818 | 2.659784 | 4.153954 | 3.267850 | 5.863294 | 1.174662 |  |
| 27 | F | 62 | T4N1M0 | 5.801536 | 3.189459 | 5.831521 | 1.277403 | 11.340770 | 1.337079 |  |
| 28 | F | 52 | T4N1M0 | 6.773105 | 2.262967 | 5.296937 | 1.294363 | 7.973147 | 1.132312 |  |
| 29 | F | 51 | T4N3M1 | 3.445137 | 1.559156 | 9.960737 | 1.304715 | 4.787405 | 1.140435 |  |
| 30 | M | 75 | T2N3M0 | 8.739682 | 3.008555 | 14.973990 | 2.392281 | 3.588896 | 0.622099 |  |
| 31 | M | 49 | T2N1M0 | 9.035586 | 4.669158 | 15.869410 | 1.252721 | 2.822488 | 0.467337 |  |
| **32** | F | 48 | **T2N1M0** | **8.272340** | **7.792920** | 5.274150 | 1.342524 | 4.963890 | 1.424073 | **Recurrence** |
| 33 | M | 54 | T2N0M0 | 11.975100 | 2.907354 | 21.879750 | 1.669649 | 6.850854 | 1.258357 |  |
| 34 | M | 46 | T2N2M0 | 1.978394 | 0.6503426 | 9.320845 | 1.649365 | 5.561047 | 1.202759 |  |
| 35 | F | 27 | T2N0M0 | 5.430947 | 2.569573 | 6.349144 | 2.514116 | 6.386770 | 1.348918 |  |
| 36 | M | 77 | T1N2M0 | 8.914005 | 2.480194 | 4.408622 | 2.296705 | 1.921472 | 0.393375 |  |
| 37 | F | 59 | T1N0M0 | 9.181534 | 2.501426 | 8.363574 | 3.576985 | 8.962563 | 2.911759 |  |
| **38** | M | 54 | **T1N2M0** | **11.446390** | **10.583210** | 8.346156 | 2.598489 | **2.474155** | **2.507035** | **Recurrence** |
| 39 | M | 71 | T1N1M0 | 16.945840 | 1.204617 | 17.132430 | 2.785711 | 1.463783 | 0.222498 |  |
| 40 | M | 51 | T1N2M0 | 9.774727 | 3.756487 | 15.770380 | 1.812505 | 4.621589 | 1.254220 |  |
| 41 | M | 34 | T4N3M1 | 11.420240 | 4.630246 | 9.146794 | 0.6560006 | 4.446640 | 1.513694 |  |
| **42** | F | 36 | **T4N2M1** | **9.264359** | **9.030859** | 11.176900 | 3.442934 | 16.255120 | 3.991229 | **Recurrence** |
| 43 | M | 38 | T4N3M1 | 14.348400 | 3.737414 | 1.238219 | 1.196300 | 10.881080 | 4.426134 |  |
| 44 | M | 50 | T4N1M1 | 12.504000 | 4.501968 | 1.589808 | 1.137758 | 2.316180 | 1.871789 |  |
| 45 | F | 41 | T4N1M0 | 16.542960 | 2.355059 | 7.395810 | 1.231367 | 1.722064 | 2.468265 |  |
| 46 | M | 31 | T4N3M1 | 11.332510 | 2.671012 | 6.602725 | 1.436133 | 6.643056 | 2.703680 |  |
| 47 | F | 37 | T3N3M1 | 11.302460 | 2.059207 | 3.287166 | 1.300393 | 10.408870 | 1.319800 |  |
| 48 | M | 32 | T3N1M1 | 10.740520 | 3.113902 | 7.313972 | 1.416660 | 3.925263 | 0.476849 |  |
| 49 | F | 32 | T3N1M0 | 6.672494 | 3.092272 | 13.325880 | 2.460692 | 1.409181 | 0.133580 |  |
| 50 | F | 28 | T2N1M0 | 10.716360 | 3.314257 | 3.193282 | 1.191993 | 8.175187 | 0.053381 |  |
| 51 | M | 44 | T2N2M1 | 6.858269 | 1.213358 | 5.442415 | 1.186800 | 16.910190 | 1.138148 |  |
| 52 | F | 39 | T2N1M0 | 4.502896 | 1.127473 | 3.9585829 | 0.9494095 | 5.442415 | 1.186800 |  |
| 53 | F | 43 | T1N0M0 | 12.302760 | 2.179737 | 7.148393 | 1.656281 | 7.088076 | 0.222207 |  |
| 54 | F | 52 | T1N1M0 | 8.778369 | 1.526363 | 2.673643 | 1.868441 | 1.981730 | 0.004482 |  |
| 55 | F | 36 | T1N1M0 | 8.987669 | 1.788592 | 8.827877 | 0.009231448 | 11.023130 | 0.2912924 |  |
| 56 | M | 38 | T1N2M0 | 5.774219 | 2.429211 | 17.074000 | 1.589504 | 15.721850 | 0.03497269 |  |
| 57 | F | 53 | T1N1M0 | 8.914568 | 2.956399 | 7.426018 | 0.5589627 | 11.041820 | 2.096023 |  |
| 58 | F | 25 | T1N2M0 | 8.309679 | 2.201890 | 1.840273 | 0.1375601 | 3.762028 | 1.428364 |  |
| 59 | M | 40 | T2N1M0 | 19.881800 | 2.744064 | 9.460293 | 1.180642 | 10.381660 | 0.5334187 |  |
| 60 | F | 42 | T2N2M1 | 7.956692 | 0.8491898 | 5.186584 | 2.188722 | 1.140817 | 0.220565 |  |
| 61 | F | 48 | T2N2M0 | 21.078570 | 0.1709829 | 7.538935 | 0.9547316 | 16.634650 | 1.876159 |  |
| 62 | M | 36 | T2N1M0 | 3.514876 | 1.356076 | 11.022820 | 0.02223442 | 6.792546 | 0.453039 |  |
| 63 | M | 49 | T2N0M0 | 20.485870 | 1.089640 | 3.411237 | 1.297778 | 15.301130 | 2.177959 |  |
| 64 | M | 42 | T3N1M0 | 5.638960 | 0.510258 | 11.338810 | 1.890688 | 14.033720 | 2.132305 |  |
| 65 | M | 52 | T3N2M1 | 9.146987 | 1.454593 | 10.766260 | 0.2108709 | 9.191110 | 0.9287143 |  |
| 66 | F | 38 | T3N1M0 | 10.462640 | 2.419579 | 10.844030 | 0.3138934 | 17.069230 | 1.581774 |  |
| 67 | M | 43 | T4N1M0 | 23.032250 | 2.932866 | 3.318219 | 0.7729172 | 8.965659 | 0.3926494 |  |
| 68 | F | 47 | T4N1M0 | 21.414180 | 1.689836 | 3.771257 | 0.5766721 | 3.394807 | 0.116086 |  |
| 69 | F | 39 | T4N3M1 | 21.714880 | 0.513833 | 7.329247 | 0.2872657 | 6.520662 | 0.7103313 |  |
| 70 | F | 38 | T4N2M1 | 15.458830 | 0.5562701 | 12.447510 | 1.164325 | 9.423391 | 0.7546807 |  |
| 71 | M | 57 | T4N3M0 | 5.746863 | 1.565037 | 2.455364 | 1.367626 | 7.282652 | 0.1019918 |  |
| 72 | M | 37 | T4N3M0 | 1.254215 | 0.04638033 | 7.451688 | 1.379642 | 12.010890 | 0.04124562 |  |
| 73 | M | 36 | T3N0M0 | 14.438750 | 2.517949 | 4.758445 | 1.769255 | 16.673130 | 0.596707 |  |
| 74 | M | 41 | T3N2M1 | 15.801230 | 1.592286 | 12.209900 | 0.3338545 | 5.272103 | 1.599318 |  |
| 75 | M | 35 | T3N0M0 | 22.374920 | 0.1348966 | 4.667443 | 1.152486 | 4.036328 | 0.4639276 |  |
| **76** | F | 20 | **T3N2M0** | **10.597970** | **12.586830** | **6.831995** | **6.068373** | **9.250784** | **10.029909** | **Recurrence and metastasis** |
| **77** | M | 59 | **T3N2M1** | **21.566278** | **22.04537457** | **13.269060** | **11.296006** | **17.937810** | **15.3905818** | **Residual lymph nodes and metastasis** |
| 78 | F | 45 | T3N1M0 | 5.914796 | 0.4540704 | 11.453420 | 1.971057 | 4.790774 | 1.662658 |  |
| 79 | M | 41 | T2N3M1 | 8.049868 | 0.4333241 | 7.483860 | 0.6587648 | 10.454460 | 0.6725692 |  |
| 80 | M | 53 | T2N0M0 | 18.112480 | 2.801604 | 8.795008 | 0.9859714 | 14.904020 | 2.215931 |  |
| 81 | F | 38 | T2N0M0 | 15.433210 | 2.086087 | 16.668360 | 0.3286138 | 2.425036 | 0.4841946 |  |
| 82 | M | 25 | T2N1M0 | 9.387019 | 0.6261955 | 1.792220 | 1.093868 | 1.883021 | 0.549541 |  |
| 83 | M | 41 | T2N1M0 | 2.951952 | 0.286506 | 5.985387 | 1.116157 | 2.563803 | 0.109758 |  |
| 84 | M | 44 | T1N2M0 | 8.117162 | 1.141947 | 6.916358 | 1.521426 | 1.820013 | 0.497692 |  |
| 85 | M | 58 | T1N0M0 | 2.069352 | 1.086065 | 6.248713 | 1.081854 | 1.520266 | 0.251029 |  |
| 86 | F | 47 | T1N3M0 | 4.145801 | 1.175421 | 9.932878 | 1.599098 | 1.933512 | 0.591090 |  |
| 87 | M | 24 | T1N0M0 | 2.31303 | 0.370883 | 9.559853 | 2.538938 | 2.623084 | 0.158540 |  |
| 88 | F | 43 | T1N1M0 | 8.202173 | 0.241310 | 7.050097 | 3.044548 | 12.223570 | 2.829780 |  |
| 89 | F | 25 | T4N2M1 | 4.297127 | 0.352295 | 8.230760 | 2.160150 | 1.863700 | 0.533642 |  |
| 90 | F | 37 | T4N1M0 | 2.149771 | 0.348892 | 4.186572 | 0.488317 | 3.225449 | 1.654227 |  |
| 91 | M | 32 | T4N3M1 | 6.969201 | 1.127836 | 10.620570 | 2.871863 | 3.210135 | 1.641625 |  |
| 92 | M | 51 | T1N1M0 | 7.109403 | 1.432578 | 10.980150 | 4.457017 | 2.352302 | 0.935713 |  |
| 93 | M | 45 | T1N0M0 | 7.915531 | 0.9062736 | 17.343920 | 13.166250 | 3.185833 | 1.621627 |  |
| 94 | M | 50 | T1N2M0 | 8.492821 | 1.581029 | 14.552290 | 2.775260 | 2.795756 | 2.300632 |  |
| 95 | M | 60 | T1N2M0 | 8.666088 | 1.783549 | 2.404168 | 0.0207947 | 1.357470 | 0.117064 |  |
| 96 | F | 42 | T3N2M1 | 7.147702 | 0.008812022 | 6.599752 | 2.452826 | 2.086721 | 1.717166 |  |
| 97 | M | 20 | T3N1M0 | 3.438286 | 1.517286 | 10.832410 | 2.367508 | 1.464688 | 0.205294 |  |
| 98 | F | 61 | T4N1M0 | 2.596659 | 0.5335665 | 11.157510 | 2.387775 | 2.618392 | 0.154679 |  |
| 99 | M | 29 | T4N1M0 | 7.252506 | 0.1313101 | 13.909790 | 5.102370 | 2.958396 | 0.434469 |  |
| 100 | M | 46 | T2N3M1 | 12.104374 | 1.127000 | 20.592790 | 7.452890 | 2.038973 | 0.677874 |  |
| 101 | F | 49 | T2N1M0 | 8.927657 | 2.089279 | 11.878370 | 3.585813 | 1.506837 | 0.239979 |  |

Patients who did not show significant decreased levels in either *MALAT1*, *AFAP1-AS1* or *AL359062* after treatment are highlighted in bold.
