# Supplementary material for: Serum long non-coding RNAs MALAT1, AFAP1-AS1 and AL359062 as diagnostic and prognostic biomarkers for nasopharyngeal carcinoma
Source: Oncotarget. 2017 Apr 13;8(25):41166–77. doi: 10.18632/oncotarget.17083 (PMC5522198; doi:10.18632/oncotarget.17083)
Supplement: Supplementary file 4 [file oncotarget-08-41166-s004.doc]

**Supplemental Table S3. Primer sequences used for qRT-PCR.**

| **lncRNAs/Genes** | **Primer sequences （5'-3'）** |
| --- | --- |
| H19 | Forward:ATCGGTGCCTCAGCGTTCGG |
|  | Reverse:CTGTCCTCGCCGTCACACCG |
| NEAT1 | Forward:CTTCCTCCCTTTAACTTATCCATTCAC |
|  | Reverse:CTCTTCCTCCACCATTACCAACAATAC |
| HNF1A-AS1 | Forward:TCAAGAAATGGTGGCTAT |
|  | Reverse:GCTCTGAGACTGGCTGAA |
| HOTAIR | Forward:CAGTGGGGAACTCTGACTCG |
|  | Reverse:GTGCCTGGTGCTCTCTTACC |
| MALAT1 | Forward:CAGTGGGGAACTCTGACTCG |
|  | Reverse:GTGCCTGGTGCTCTCTTACC |
| LINCRNA-P21 | Forward:GGGTGGCTCACTCTTCTGGC |
|  | Reverse:TGGCCTTGCCCGGGCTTGTC |
| GAS 5 | Forward:CTTCTGGGCTCAAGT GATCCT |
|  | Reverse:TTGTGCCATGAGACTCCATCAG |
| AFAP1-AS1 | Forward:AATGGTGGTAGGAGGGAGGA |
|  | Reverse:CACACAGGGGAATGAAGAGG |
| LET | Forward:CCTTCCTGACAGCCAGTGTG |
|  | Reverse:CAGAATGGAAATACTGGAGCAAG |
| LOC401317 | Forward:AGTCCAACGTGGTCCCTTC |
|  | Reverse:AACTTTCTCCGGGGGTTC |
| NAG7 | Forward:GCGTGTGTGTGTGTATTCTTTC |
|  | Reverse:AGCTTGGAGCTATGCTTGTT |
| CCAT2 | Forward:CCCTGGTCAAATTGCTTAACCT |
|  | Reverse:TTATTCGTCCCTCTGTTTTATGGAT |
| LOC84740 | Forward:TCGCTCAATGGAGTGACGGCA |
|  | Reverse:CGGCTGAGACCGCTGAGAACTT |
| ENST00000498296 | Forward:GTGCTATTGCCCCATGTGAA |
|  | Reverse:TGATGCCGAAAACCTGTAAGAA |
| AL359062 | Forward:CAATGGTGCTAATCTCAGACCTAAA |
|  | Reverse:TCAAAAGGGAAATACAGGCACA |
| ENST00000438550 | Forward:AGCAAGTGGAATGAAAGAACAGAAC |
|  | Reverse:ACCTCTTCTATTGCCAGAACCAAC |
| AF086415 | Forward:AGCGCGACTTCTCTGTCTCT |
|  | Reverse:GCAGAGGAGGAGACGCTGA |
| AK095147 | Forward:ACGAGTGACCGAAGCTGAAC |
|  | Reverse:GCACCATCCAGAGGGATTTA |
| RP1-179N16.3 | Forward:CGCGTTAGGAGATTCTGGAG |
|  | Reverse:AGGGTGGATACAGGCTCCTT |
| MUDENG | Forward:ACTTTGTGGCACCGTGAGAT |
|  | Reverse:GGCCCACTAAATGCAGAGTC |
| AK056098 | Forward:GGCCTCGGGGTAGAACTTAC |
|  | Reverse:CAAGCCTCCTGGTCTTTCTG |
| AK294004 | Forward:CAAGCCTCCTGGTCTTTCTG |
|  | Reverse:CAAGCCTCCTGGTCTTTCTG |
| LNC-C22ORF32-1 | Forward:TGCTCATCTTCTGCCACAGT |
|  | Reverse:AGGGCAGTGATGAGGAACC |
| LNC-ZNF674-1 | Forward:AGCACTTGGCCCTAAAGAGA |
|  | Reverse:AACATACTGGCCCAAACAGC |
| LNC-TLR4-1 | Forward:CCACACAAATGGGCAAGAAT |
|  | Reverse:GCAAAATCCTGAAGGTTCAAA |
| LNC-BCL2L11-3 | Forward:AGCAGATGCTGTGCCTGATA |
|  | Reverse:CCTTTCTCGACCCAGAAGC |
| LNC-AL355149.1-1 | Forward:GAAAACTAGGCGTCTGGGAAC |
|  | Reverse:CAAACAATGGGAGCAAGTCC |
| ENST00000411815 | Forward:ACCTGAGCCTCCCAAAGTTC |
|  | Reverse:TGAGCCAATGATTAAAGACAGC |
| ENST00000429469 | Forward:AGAAAGACTCCTGCCAAACCT |
|  | Reverse:ATGTGCCACTCTGTCCCCA |
| ENST000000440518 | Forward:TTGGCAAGAAAGTTGGAGATG |
|  | Reverse:GGCAGCAGTGAGACAGTGAAT |
| ENST00000443373 | Forward:GGTCCTTTGGAATCCTCCTACT |
|  | Reverse:CAGTAATCATAGATGGTAT |
| ENST00000457799 | Forward:AGGAAACAGCGAGTTAGCA |
|  | Reverse:GTTGGGAGGTGATTGGATT |
| ENST00000513638 | Forward:GCTACTGCCAAGTCTGGTCAC |
|  | Reverse:CAGATAATACCTGCCTTGAAATG |
| ENST00000514571 | Forward:TGGATGCAATGCAACGGTA |
|  | Reverse:GGGACTGAGGTGGGAGGAT |
| ENST00000565929 | Forward:CATACCAGGCACAGAAACATACAA |
|  | Reverse:CTGTCTCCCACTCTTCTTCCTCA |
| ENST00000566575 | Forward:CTCTGCCTGGCTATTTGCTC |
|  | Reverse:TGTCACTTGACCTCACCCTGT |
| UC010ZYE | Forward:CAGGGCAGGTGTTTCATCC |
|  | Reverse:CTGGGCATTGTCTTGGTTGT |
| LINC00592 | Forward:TGAATGGGAAGAAATGGGTT |
|  | Reverse:GATGCAAGTGGCAGACAAAG |
| GAPDH | Forward:TGTGTTGGCGTACAGGTCTTTG |
|  | Reverse:GGGAAATCGTGCGTGACATTAAG |
| LMP1 | Forward:GGACTGGACTGGAGGAGCC |
|  | Reverse:GAGGGAGTCATCGTGGTGGTG |
| EBNA1 | Forward:GGACCCGGCCCACAACCTG |
|  | Reverse:CTACTCCCACTCCTTCCCGTCCTC |
| CDH1 | Forward: TGAAGCCCCCATCTTTGTGC |
|  | Reverse: GGCTGTGTACGTGCTGTTCT |
| CDH2 | Forward: ATCCTACTGGACGGTTCG |
|  | Reverse: TTGGCTAATGGCACTTGA |
| MMP2 | Forward: CATTTGGCGGACTGTGAC |
|  | Reverse: GGGTGCTGGCTGAGTAGAT |
| MMP3 | Forward: CAGGAGAGAGCAGAAACC |
|  | Reverse: CACTCCCAGGCTTTCTAC |
| MMP9 | Forward: TCCACCCTTGTGCTCTTCC |
|  | Reverse: GCCACCCGAGTGTAACCAT |
| CDKN1B | Forward: TCCAAGAGGAAGCCCTAATCC |
|  | Reverse: ACAAACTGAGACTAAGGCAGAAGATG |
| CCND1 | Forward: GTCATTCTTCCACGATCTTTGTAGTC |
|  | Reverse: TCAATCCTGGACACAAAGTGTTATC |
| CCNE1 | Forward: GTCTACCTGGAACAAGAAGAGAGATTC |
|  | Reverse: TCAATCCTGGACACAAAGTGTTATC |
| CDK2 | Forward: CATGTTGACCGAGCTGGAGAA |
|  | Reverse: TCCCTGTAGACGGCATGGA |
| CDK4 | Forward: CACAGCCACCTAAACGGTTCA |
|  | Reverse: CCAAGGCCACCAGCTTCTAG |
